# Logistics–client stable matching under 4PL to reduce the empty-loaded rate

**DOI:** 10.1371/journal.pone.0308951

**Published:** 2025-03-19

**Authors:** Jian Jiang, Jie Li, Boyuan Xia

**Affiliations:** 1 School of Computer and Electrical Engineering, Hunan University of Arts and Science, Changde, China; 2 College of Systems Engineering, National University of Defense Technology, Changsha, China; MIT World Peace University Faculty for Engineering and Technology, INDIA

## Abstract

Logistics, as a tertiary industry, has developed rapidly and become an important part of the national economy. However, owing to the behindhand logistics pattern, the logistics vehicles drive empty-loaded on their return trip, resulting in wastage of half of the delivery resources. This paper proposes a logistics–client matching model under fourth-party logistics (4PL) to reduce the empty-loaded rate. First, a preference calculation model between logistics providers and clients was constructed. Next, for clients with small quantities of goods, a linear logistics−client one-to-one stable matching model was constructed based on the stable marriage matching model. Then, for clients with large quantities of goods, a linear logistics−client many-to-one stable matching model was constructed with a novel linear many-to-one stable matching constraint. Finally, the case study indicated that the linear model supports large-scale and global optimization. The real case study verified that the proposed stable matching model is effective in reducing the empty-loaded rate compared to the ordinary matching model, and the matching solution was fairer.

## 1 Introduction

Recently, online retailers have become increasingly important in the retail business, resulting in numerous transportation requirements for the products sold online. In conventional transportation, particularly when an enterprise has its own logistics system, many transportation vehicles must return to the initial site with empty or minimal load. In China, the transportation efficiency needs significant improvement because of the high empty-loaded rate of 40% in the conventional transportation industry [1]. A high empty-loaded rate causes unnecessary costs in the logistics industry and occupies insufficient road resources. Vehicles with untapped capacity can provide logistics services at a lower price. Further, many small enterprises do not possess their own logistics systems but hire transportation services from the open market. Therefore, it is important to study the mechanism and method for effectively reducing the empty-loaded rate of the logistics industry to facilitate the development of green and smart logistics.

The bidirectional requirements spawned the generation of fourth-party logistics (4PL), which was first proposed by Accenture in 1998. The 4PL provides a complete set of supply chain solutions through its information technology, integration ability etc., which can incur profits. It can help enterprises reduce costs and effectively integrate resources [2]. Nordic Car Logistic, the largest vehicle logistics provider in Sweden, has been trying to be a 4PL provider in its industry since 2017 [3].

Stability is the priority for the 4PL platform as an unstable matching pair induces the user to break the current matching to form a new deal with other users, thus triggering a chain of dishonest actions [4]. The 4PL platform must ensure the stability of the assignment scheme such that no user can unilaterally break the existing match balance. Therefore, the main focus of this study is to form stable assignments between logistics vehicles and clients.

In literature, stability and fairness are seldom considered in the logistics−client assignment of the 4PL platform. Most studies consider the maximization of the total satisfaction of both sides as their objective. Certain studies add constraints for the satisfaction deviation of any matched logistics and client to be smaller than a threshold value. In this study, two main constraints were established to guarantee stability: (1) stable matching constraints were added to the one-to-one logistics−client assignment problem, and (2) improved linear stable matching constraints were designed for the many-to-one logistics−client assignment problem.

This study makes several key contributions: (1) Two stable matching models were developed for the one-to-one and many-to-one logistics-client assignment problems. The first model was designed for situations where clients have small delivery requirements, while the second model was tailored for scenarios involving clients with bulk delivery needs. (2) The linearity of the two proposed models ensures their suitability for solving large-scale optimization problems. (3) A real-world case study was conducted to demonstrate the effectiveness of the proposed model in reducing the empty-loaded rate and minimizing preference deviations between the involved parties.

The remainder of this paper is organized as follows. Section 2 briefly reviews relevant research related to 4PL planning and stable matching. Section 3 describes the basic problem and defines the required notations. Section 4 presents the mathematical models of the logistics−client one-to-one and many-to-one stable matching problems. An example and a real case study were analyzed using the proposed model to illustrate its effectiveness. Finally, Section 7 summarizes the study and presents scope for future work.

## 2 Literature review

This section introduces the research on the planning, optimization, and decision-making of 4PL, one-to-one matching, many-to-one matching, stable matching, and fair matching in the field of operations research.

### 2.1 4PL research

In 1998, the American Accenture company proposed 4PL to provide information support, supply chain management, transportation planning, logistics consulting, etc. for 1PL, 2PL, and 3PL. 1PL refers to the logistics activities organized by sellers, producers, or suppliers [5]. The main business of these organizations is the production and supply of goods, and the investment, operation, and management of logistics networks and equipment. The 2PL activity in the supply chain refers to distributors who deliver purchased goods. For example, wholesalers pick up goods from factories, deliver goods to retail stores or clients, establish their logistics and distribution network, maintain inventory, etc., all of which refer to the 2PL activities. 3PL refers to an enterprise with substantial logistics tools that provide logistics-related services, such as transportation, warehousing, inventory management, order management, information integration, etc., to other companies to provide complete services.

The difference between the aforementioned logistics mode and 4PL is that the latter is a highly information-based and coordination-based platform that is advantageous to the logistics providers and clients with delivery demands. There is no overlapping relationship between 4PL and product providers, consumers, and 3PL. 4PL aims to provide complete logistics supply chain solutions through strong information integration and sharing capabilities, to effectively integrate logistics resources, reduce costs, and avoid resource waste [6–9]. According to the concept and identification standard, Frost and Sullivan argued that the 4PL market has witnessed considerable revenue growth, from approximately 4.7 billion euros in 2002 to approximately 13 billion euros in 2010 [10]. Several studies mainly focus on the role in the supply chain, operational advantages, leading factors, structure design and optimization decision model of the 4PL [11–17]. The planning, optimization, and decision-making research is important to achieve maximum performance, and research on this aspect accounts for the largest proportion of the entire research scope of the 4PL.

Tao et al. modeled the route planning problem in 4PL as a mixed-integer programming model and proposed a column generation approach combined with a graph search heuristic to efficiently solve the model. Tao et al. studied the route planning problem in fourth party logistics [18]. Huang et al. studied a fourth-party logistics routing optimization problem (4PLROP) with uncertain delivery times under emergency conditions [19]. Based on the uncertainty theory, they proposed a novel 4PLROP uncertain programming model (UPM) under emergency conditions by describing the uncertain delivery time as an uncertain variable. Considering the multipoint-to-multipoint 4PL routing optimization problem, Li et al. proposed a multitask routing optimization mathematical model with reliability constraints and solved the problem by using a messy genetic algorithm, the result of which proved to be the same as that of the enumeration algorithm [20]. Lentz proposed a 4PL leagility framework by defining nine types of leagilities [21]. They concluded that 4PL providers can improve flexibility within a supply network based on their expertise in coordinating and integrating virtual supply chains and transportation networks. Schramm et al. investigated the potential future of 4PL and concluded that 4PL would transform the simple organized transportation activity to IT-based value-added service activities [22]. From the perspective of the fourth party logistics, Haproposed a supply chain network design problem considering the psychological behavior of customers [23].Yuan investigated a new transportation services procurement problem for 4PL, which involves three features: the 4PL’s loss-averse behavior, price and non-price attributes, and multiple transportation requests [24].

### 2.2 One-to-one matching problem

The one-to-one matching problem originates from the stable marriage matching problem proposed by Gale and Shapley [25]. Vate first defined the mathematical symbol of preference ranking and solved the stable marriage matching problem using a mathematical programing model [26]. Teo and Sethuraman used the structural properties of the fractional solution to express the stable marriage matching problem through a geometric approach and used a 2-approximation algorithm to solve the proposed new linear programing model [27].

The stable matching mechanism proposed by the GS algorithm of Gale and Shapley is biased, that is, it is the best stable matching for one side and worst for the other side [25]. Therefore, the problem of stable matching should be solved fairly. Gusfield and Irving established the concept of fairness based on stable marriage matching to avoid the phenomenon of one side being extremely good and the other being extremely poor [28]. Knuth and DE proposed a minimum regret matching method to achieve matching fairness on both sides [29]. Klaus and Klijn analyzed three probabilistic stable matching mechanisms based on matching fairness: (1) the lottery employment mechanism proposed by Aldershof, Carducci, and Lorenc, (2) the random ranking mechanism proposed by Ma, and (3) the fair random order mechanism proposed by Romero−Medina [30–33]. Their analysis showed that these three mechanisms may produce completely different results, and the probability distributions of stable matching sets are not necessarily consistent. They further proved that random matching mechanisms do not necessarily produce stable matching schemes.

In addition to the marriage problem, one-to-one matching has also been applied to many other domains. The kidney donation system designed by Roth, Sönmez, and Ünver could overcome several limitations of kidney donation and maximize the matching rate of kidneys [34]. Their research on the matching of kidney donations has had a huge impact on the development of organ transplantation in the medical community and won the Nobel Prize in Economics. Wang, Agatz, and Erera considered the concept of stability for the first time based on shared-ride and established a mathematical programing method for the stable or nearly stable matching model [35]. Li et al. used a simple and effective stable matching (STM) model to coordinate the selection process using a decomposition-based multi-objective evolutionary algorithm (MOEA/D) [36]. Kong, Jiang, and Liang proposed the consideration of fairness in one-to-one matching, built a multi-satisfaction model to obtain the highest possible fair and stable matching scheme, and used a multi-objective genetic algorithm to solve the problem [37]. Kasajima considered two-sided one-to-one matching problems (between men and women) and studied a new requirement called “own-side singles monotonicity” [38]. Gualdani studied partial identification of the preference parameters in models of one-to-one matching with perfectly transferable utilities, without imposing parametric distributional restrictions on the unobserved heterogeneity and with data on one large market [39].

### 2.3 Many-to-one matching problem

Many-to-one matching is a conventional problem in bilateral matching field. It originated from the college admission problem proposed by Gale and Shapley [25]. Baiou and Balinski transformed the college admission problem into a many-to-one matching problem and proposed a new many-to-one stable matching model [40]. Subsequently, many scholars have studied the many-to-one matching problem and obtained many theoretical and practical results.

Dur and Ikizler proposed an asymptotically stable calculation algorithm for the best many-to-one stable matching of a school recruiting a student [41]. Romero−Medina and Triossi studied two sequential admission mechanisms under the framework of many-to-one matching, which allowed students to send multiple applications to multiple schools [42]. These two mechanisms were consistent with the actual situation of the school enrollment process and realized the Nash equilibrium of a perfectly stable matching set of subgames. Martínez et al. proved that the blocking lemma applies to many-to-one matching models [43]. Bando designed a modified deferred acceptance algorithm to address this problem [44]. The algorithm found an optimal quasi-stable matching of the workers. Rasulkhani and Chow proposed a many-to-one matching between users and operators to evaluate a transportation system composed of operators and users [45]. Cesco used game theory to associate the many-to-one matching problem with a hedonic game [46].

Considering the applications of the problem, the many-to-one stable matching model is widely used in the contexts where both sides possess the selection right and desire for fairness. Recently, with the development of economy and society, the many-to-one matching research has been explored from the perspectives of conventional universities−student matching, hospitals−intern matching in certain emerging fields, such as commodity−transactions matching, service supply−demand matching in IT, personnel−position matching in human resources management, and network user−communication resources matching in wireless communication network [47–49]. Assila, Kobbane, and El adopted a many-to-one stable matching game to achieve the low-latency exploiting fogs and caching in the 5G Internet of things [48]. Liu et al., Revilla, and Bando concretely studied the many-to-one matching in a talent market, where each agent’s preferences depend on the performance of companies and decisions of other agents [50–52]. Liu et al. adopted the many-to-one stable matching algorithm to minimize user terminals in the low earth orbit satellite networks [50]. Chen, Gravin, and Lu studied the mechanism design question without payment via many-to-one stable matching theory [53]. Huang developed an integer programming approach on two-sided many-to-one matching by investigating stable integral matchings of a fictitious continuum market induced from the original matching market [54]. In a many-to-one matching model with responsive preferences in which indifferences are allowed, Agustín studied three notions of core, three notions of stability, and their relationships [55].

A basic premise of the many-to-one stable matching method is that each member in party A requires only one member of party B. Each member in party B requires a fixed number of $q_{i}$ in party A, where *i* is the corresponding subscript of a member in party B. However, in the context of this study, $q_{i}$ cannot be fixed because the number of logistics providers a contractor can hire depends on the resource requiremec distribution networks. Few researchers have studied the optimization of logistics task matching; however, no study has applied empty-loaded truck distribution to the 4PL scheme. This study was aimed at solving the problem of the empty return of logistics vehicles while effectively avoiding the wastage of social resources. This research can help alleviate traffic congestion and promote the sustainable development of ecology and energy.

## 3 Problem description

**Fig 1:**
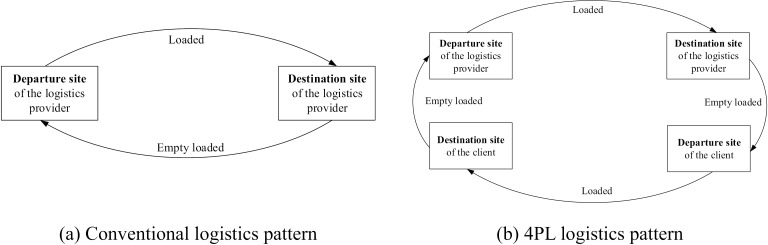
Research background. The 4PL logistics pattern can be divided into four routes. Route 1 starts from the departure site of the logistics provider to the destination of the logistics provider, with a loaded logistics vehicle. In this study, Route 1 was not considered, because the route and transportation task are predetermined. Route 2 starts from the original logistics destination to the client’s departure site, with an empty logistics vehicle. Route 3 starts from the client’s departure site to the client’s destination; the client’s goods are delivered by the logistics vehicle. Route 4 starts from the client’s destination to the original departure site of the logistics provider, with an empty logistics vehicle. To reduce the empty-loaded rate, the 4PL platform should minimize the distance of routes 2 and 4 by matching appropriate clients to the logistics vehicles under stable constraints.

Generally, vehicles of self-owned logistics enterprises return empty loaded after the delivery of goods, as shown in Fig 1(a). As a solution provider, the 4PL is an intermediate service platform that assigns appropriate clients with delivery demands to logistics vehicles during the return trip of the vehicles to reduce the empty-loaded rate, as shown in Fig 1(b).

Defining the appropriateness of a matching pair between a logistics vehicle and client is a two-fold question. For a matching pair, the logistics and client parts have their respective perspectives that are termed as “preference factors”. The preference factors of the logistics provider with respect to the client are the empty-loaded rate, load factor [51], and profit ratio while the preference factors of the client with respect to the logistics provider are the cost savings, waiting time, and service quality. Therefore, the problem can be preliminarily described as maximizing the overall preferences of the logistics provider and client with the satisfaction of matching stability and certain other basic constraints and assumptions.

The variable notations used in the study are listed in Table 1, where *i* represents the logistics part and *j* represents the client part.

**Table 1:** Variable notations.

Indices	Implications
m	Number of logistics providers, *i* = {1, 2, …, *m*}
n	Number of clients, *j* = {1, 2, …, *n*}
Decision variables	
$x_{ij}$=1/0	Matching variable of logistics provider *i* and client *j*
Parameters	
$W_{i}$	Maximum weight that the logistics provider’s vehicle *i* can carry
$V_{i}$	Maximum volume that the logistics provider’s vehicle *i* can carry
$W_{j}^{'}$	Total weight of goods of client *j*
$V_{j}^{'}$	Total volume of goods of clients *j*
$b_{j}^{'}$	Market price in the local region of client *j*/unit weight/unit distance
$b_{i}$	Charge of logistics *i*/unit weight/unit distance
$c_{i}$	Transporting cost of logistics *i*/unit weight/unit distance
$v_{i}$	Average travel speed of logistics provider *i*
$d_{i1}$	Distance of route 1
$d_{ij2}$	Distance of route 2
$d_{j3}$	Distance of route 3
$d_{ij4}$	Distance of route 4
$s_{i}$	Average appraisement score (1 − 5) of logistics *i* in the 4PL platform
$pe_{ij}$	Preference value on the empty-loaded rate of logistics provider *i* to client *j*
$pf_{ij}$	Preference value on the load factor of logistics provider *i* to client *j*
$pp_{ij}$	Preference value on the profit ratio of logistics provider *i* to client *j*
$pw_{ji}$	Preference value on waiting time of client *j* to logistics provider *i*
$ps_{ji}$	Preference value on service quality of client *j* to logistics provider *i*
$pc_{ji}$	Preference value on cost-saving of client *j* to logistics provider *i*
$A=\left[a_{ij}\right]$	Preference matrix of logistics providers to clients
$B=\left[b_{ji}\right]$	Preference matrix of clients to logistics providers

## 4 Mathematical model

This section introduces the problem of high empty-loaded rate of transport vehicles for logistics providers. First, it describes certain factors that affect the empty-loaded rate and uses the TOPSIS method to address these factors. Second, we built a mathematical model to solve the problem. Finally, we analyzed the obtained results.

### 4.1 Calculation of preference factors

The three preference factors of logistics providers with respect to clients are the empty-loaded rate, load factor, and profit ratio. The specific calculation methods are as follows:

(1) Empty-loaded rate represents the proportion of the overall length with respect to the empty loaded driving length of a logistics vehicle, as shown in (1). The $d_{i1}$ and $d_{j3}$ are fixed. The empty-loaded rate preference value, $pe_{ij}$, is influenced by $d_{ij2}$ and $d_{ji4}$. The two values depend the client assigned by the 4PL platform to the logistics vehicle.


$$pe_{ij}=\frac{\left(d_{ij2}+d_{ji4}\right)}{d_{i1}+d_{ij2}+d_{j3}+d_{ji4}}$$ (1)

(2) Load factor represents the proportion of the vehicle load capability with respect to the carried goods. The load factor preference value depends on the weight and volume of the client goods and vehicle load capacity of the logistics vehicle, as shown in (2). The logistics vehicle may carry maximum goods within the load capacity to increase the load factor.


$$pf_{ij}=\max\left\{\frac{W_{j}{}^{'}}{W_{i}},\frac{V_{j}{}^{'}}{V_{i}}\right\}$$ (2)

(3) Profit ratio represents the revenue per unit distance under the matched delivery task, as shown in (3). A logistics part accepts a task with a higher profit ratio.


$$pr_{ij}=\frac{\left(b_{i}-c_{i}\right)d_{j3}W_{j}{}^{'}-c_{i}\left(d_{ij2}+d_{ji4}\right)}{d_{ij2}+d_{j3}+d_{ji4}}$$ (3)

The preference of the client with respect to logistics providers also includes three indices: waiting time, service quality, and cost saving. Their respective calculation method is as follows:

(1) Waiting time refers to the time that a client waits for a logistics vehicle to arrive at the transportation departure site of the client, as shown in (4). It depends on the distance 2 and average speed of a logistics vehicle. Clients hope to enhance transportation efficiency; the lower the waiting time, higher is the preference value.


$$pw_{ji}=\frac{d_{ij2}}{v_{i}}$$ (4)

(2) Service quality refers to the average scores of a logistics provider in historical delivery orders provided by the 4PL platform. The calculation formula is shown in (5), where 5 is the maximum appraisal score to scale the service quality in the range of [0, 1].


$$ps_{ji}=\frac{s_{i}}{5}$$ (5)

(3) Cost saving refers to the saved cost of a client whose goods are delivered by the matched logistics provider, when compared with the average market cost, as shown in (6).


$$pc_{ji}=\left(b_{j}{}^{'}-b_{i}\right)d_{j3}W_{j}{}^{'}$$ (6)

### 4.2 TOPSIS based preference matrix calculation

In this study, we used the TOPSIS method, a multi-attribute evaluation method, to aggregate preference factors to a preference matrix between logistics providers and clients. The TOPSIS method evaluates the Euclidean distance between the assessment point and positive and negative ideal points. The closer the assessment point to the positive ideal point and farther to the negative ideal point, the better is the evaluation.

Here, we consider the preference matrix of logistics providers with respect to clients as an example to illustrate the specific steps to obtain the preference matrix. We assume the preference matrix of logistics providers to clients be $A={\left[a_{ij}\right]}_{m\times n}$. We further assume the empty-loaded rate preference matrix be $PE={\left[pe_{ij}\right]}_{m\times n}$, load factor matrix be $PF={\left[pf_{ij}\right]}_{m\times n}$, and profit ratio preference matrix be $PP={\left[pp_{ij}\right]}_{m\times n}$.

Step 1: Normalization.

Assuming the normalized matrices of PE, PF, and PR be $PE^{\prime }={\left[pe_{ij}{}^{'}\right]}_{m\times n}$, $PF^{\prime }={\left[pf_{ij}{}^{'}\right]}_{m\times n}$, and $PP^{\prime }={\left[pp_{ij}{}^{'}\right]}_{m\times n}$, as computed in (7).


$$pe_{ij}{}^{'}=\frac{pe_{ij}}{\max\left\{PE\right\}},pf_{ij}{}^{'}=\frac{pf_{ij}}{\max\left\{PF\right\}},pr_{ij}{}^{'}=\frac{pp_{ij}}{\max\left\{PP\right\}}$$ (7)

Step 2: Defining the positive ideal point $x^{+}$ and negative ideal point $x^{-}$, as shown in (8).


$$x^{+}=\left(\min\left\{PE^{\prime }\right\},\max\left\{PF^{\prime }\right\},\max\left\{PR^{\prime }\right\}\right)$$ (8)

$$x^{-}=\left(\max\left\{PE^{\prime }\right\},\min\left\{PF^{\prime }\right\},\min\left\{PR^{\prime }\right\}\right)$$ (9)

Step 3: We calculate the distance from each point to the positive and negative ideal points $s_{ij}^{+}$, $s_{ij}^{-}$, as shown in (10) and (11).


$$s_{ij}^{+}=\sqrt{{\left(pe_{ij}{}^{'}-\min\left\{PE^{\prime }\right\}\right)}^{2}+{\left(pf_{ij}{}^{'}-\max\left\{PF^{\prime }\right\}\right)}^{2}+{\left(pr_{ij}{}^{'}-\max\left\{PR^{\prime }\right\}\right)}^{2}}$$ (10)

$$s_{ij}^{-}=\sqrt{{\left(pe_{ij}{}^{'}-\max\left\{PE^{\prime }\right\}\right)}^{2}+{\left(pf_{ij}{}^{'}-\min\left\{PF^{\prime }\right\}\right)}^{2}+{\left(pr_{ij}{}^{'}-\min\left\{PR^{\prime }\right\}\right)}^{2}}$$ (11)

Step 4: We calculate the comprehensive appraisal value, that is, the comprehensive preference of a logistics provider with respect to a client, as shown in (12).


$$a_{ij}=\frac{s_{ij}^{-}}{s_{ij}^{-}+s_{ij}^{+}}$$ (12)

Then, we obtain the preference matrix *A* of logistics providers with respect to clients and similarly, the preference matrix $B={\left[b_{ji}\right]}_{n\times m}$ of clients with respect to logistics providers.

### 4.3 One-to-one stable and fair matching model

For small quantities of goods, a single logistics vehicle is sufficient to satisfy delivery demands. Here, based on the preferences of the logistics provider and client, a one-to-one stable matching model of logistics providers and clients is constructed as follows:


$$\text{maximize}\;z_{1}=\overset{m}{\underset{i=1}{\sum }}\overset{n}{\underset{j=1}{\sum }}x_{ij}a_{ij}$$ (19)

$$\text{maximize}\;z_{2}=\overset{m}{\underset{i=1}{\sum }}\overset{n}{\underset{j=1}{\sum }}x_{ij}b_{ji}$$ (20)

$$\text{minimum}\;z_{3}=\overset{m}{\underset{i=1}{\sum }}\overset{n}{\underset{j=1}{\sum }}x_{ij}\left|a_{ij}-b_{ji}\right|$$ (21)

$$\text{s}\text{.t}\text{.}\;\overset{m}{\underset{i=1}{\sum }}x_{ij}\leq 1,\forall j$$ (22)

$$\overset{n}{\underset{j=1}{\sum }}x_{ij}\leq 1,\forall i$$ (23)

$$x_{ij}\left(W_{i}-W_{j}{}^{'}\right)\geq \text{0, }\forall i,j$$ (24)

$$x_{ij}\left(V_{i}-V_{j}{}^{'}\right)\geq \text{0, }\forall i,j$$ (25)

$$x_{ij}\left[\left(b_{i}-c_{i}\right)d_{j3}W_{j}{}^{'}-c_{i}\left(d_{ij2}+d_{ji4}\right)\right]\geq \text{0, }\forall i,j$$ (26)

$$x_{ij}\frac{\left(d_{ij2}+d_{ji4}\right)}{d_{i1}+d_{ij2}+d_{j3}+d_{ji4}}\leq \gamma \text{, }\forall i,j$$ (27)

$$x_{ij}\left(b_{j}{}^{'}-b_{i}\right)\geq 0,\forall i,j$$ (28)

xij+∑j′<jixij′+∑i′<ijxi′j≤1,∀i,j (29)

$$x_{ij}\in \left\{0,1\right\},\forall i,j$$ (30)

The first objective function (19) maximizes the overall preference of logistics providers with respect to clients.

The second objective function (20) maximizes the overall preference of clients with respect to logistics providers.

The third objective function (21) minimizes the overall preference deviation to achieve fairness.

Constraints (22) show that one client can only match with one logistics provider.

Constraints (23) represent that one logistics provider can only match with one client.

Constraints (24) show that the weight of the client’s goods should not exceed the maximum loading weight of the matched logistics vehicles.

Constraints (25) indicate that the volume of the client goods must be less than the maximum load volume of the matched logistics vehicles.

Constraints (26) signify that the profit ratio of logistics providers should be greater than zero.

Constraints (27) show that the empty-loaded rate of logistics providers is not more than *γ*.

Constraints (28) indicate that the cost savings of clients should be greater than zero.

Constraints (29) represent stable matching to prevent blocking pairs.

Constraints (30) show that the value of the decision variable is 0 or 1, which means that logistics provider *i* is not matched or matched with client *j*.

For an easy solution, this study converts the three objective functions of the aforementioned model to a single-objective optimization model, as shown in (32).


$$\text{maximum}\;\overset{m}{\underset{i=1}{\sum }}\overset{n}{\underset{j=1}{\sum }}x_{ij}a_{ij}b_{ji}$$ (32)

It considers the minimization of preference deviation. For any two possible values $a1_{ij},b1_{ji}$ and $a2_{ij}\text{, }b2_{ji}$ of the variables $a_{ij},b_{ji}$ meeting $a1_{ij}+b1_{ji}=a2_{ij}+b2_{ji}$, it can be proved that $0<a1_{ij}b1_{ji}<a2_{ij}b2_{ji}$ when $\left|a1_{ij}-b1_{ji}\right|>\left|a2_{ij}-b2_{ji}\right|$.

### 4.4 Many-to-one stable and fair matching model

To satisfy the transportation demand to deliver large quantities of goods for clients, it is necessary to match multiple logistics vehicles to provide sufficient transportation services. First, we assume that each logistics vehicle delivers goods at its maximum load unless the weight of the remaining goods of a client is less than the maximum load of the logistics vehicle. Moreover, the preference elements between the logistics providers and clients in this many-to-one problem are the same as those in the one-to-one problem.

The main objective is to propose new many-to-one stable matching constraints. In a conventional college admission many-to-one stable matching problem [23], the stable matching constraints are defined as follows:


$$\overset{a}{\underset{i=1}{\sum }}x_{ij}\leq 1,\forall i\in \left\{1,2,...,a\right\}$$ (34)

$$\overset{u}{\underset{j=1}{\sum }}x_{ij}\leq q_{j},\forall j\in \left\{1,2,...,u\right\}$$ (35)

$$q_{j}x_{ij}+q_{j}\underset{i^{\prime }{>}_{j}i}{\sum }x_{i^{\prime }j}+\underset{j^{\prime }{>}_{i}j}{\sum }x_{ij^{\prime }}\geq q_{j}\text{, }\forall i\in \left\{1,2,...,a\right\},\forall j\in \left\{1,2,...,u\right\}$$ (36)

$$x_{ij}=\left\{0,1\right\},\forall i\in \left\{1,2,...,a\right\},j\in \left\{1,2,...,u\right\}$$ (37)

Constraints (34) show that each student can only be admitted to one school.

Constraints (35) indicate that the number of students matched to each school should not exceed the limited enrollment number.

Constraints (36) guarantees stable matching.

Constraints (37) show that the value of the decision variable is 0 or 1.

However, in the many-to-one logistics−client stable matching problem, unlike the conventional college admission many-to-one matching, clients have no fixed required amount of logistics vehicles because the maximal loads of different logistics vehicles vary. The requirement is satisfied until the matched logistics vehicles can carry all the goods of the client. Therefore, we cannot determine the value of $q_{j}$ in the many-to-one stable matching constraints in (36).

In this study, we established an appropriate improvement based on the conventional many-to-one stable matching constraints. First, when a matching pair (*i*, *j*) is defined as a blocking pair, it is concluded that the following three conditions are achieved simultaneously:

There is no matching between logistics provider *i* and client *j*, that is, $x_{ij}=0$.
Logistics provider *i* believes that its matched client is worse than client *j*, that is, ∑j′<jixij′=1. Then, logistics provider *i* prefers to match with client *j*.
For client *j*, we assume a total load of vehicles of logistics providers that are better than logistics provider *i* be $\underset{i^{\prime }{>}_{j}i}{\sum }x_{i^{\prime }j}W_{i^{\prime }}$. The value does not exceed the total weight of goods of client *j* after adding the load capacity of the logistics provider *i*, that is, $\underset{i^{\prime }{>}_{j}i}{\sum }x_{i^{\prime }j}W_{i^{\prime }}+W_{i}<W_{j}{}^{'}$. Then, client *j* has the willingness to replace any matched logistics providers that is worse than the logistics provider *i* (if there are no other providers who are worse than provider *i*, client *j* is willing to form a new matching with provider *i*).

Next, the aforementioned logical constraints are converted into linear constraints, as shown in (38).


xij+1−∑j′<jixij′+∑i′>ijxi′jWi′+Wi−Wj'Wj'≥0, ∀i,j (38)

The rationality of the new constraints for the logistics−client many-to-one stable matching is proved as follows:

*Proof 1*.
For any matching pair (*i*, *j*):

Let *a*, *b*, and *c* represent $x_{ij}$, 1−∑j′<jixij′, and $\frac{\left(\sum_{i^{\prime }{>}_{j}i}x_{i^{\prime }j}W_{i^{\prime }}+W_{i}-W_{j}{}^{'}\right)}{W_{j}{}^{'}}$, respectively. Let flag=a+b+c. The value set of *a* is {0, 1}, value set of *b* is {0, 1}, and value range of *c* is $\left(-1,+\infty \right)$. All possible values of all variables are listed in Table 2.

**Table 2:** Variables enumeration table.

Variables *a*	Variables *b*	Variables *c*	Constraint values flag=a+b+c	Blocking pair or not
1	0	−1<c<0	0<flag<1	N
1	0	0≤c	1≤flag	N
1	1	−1<c<0	1<flag<2	N
1	1	0≤c	2≤flag	N
0	0	−1<c<0	−1<flag<0	Y
0	0	0≤c	0≤flag	N
0	1	−1<c<0	0<flag<1	N
0	1	0≤c	1≤flag	N

If $x_{ij}=1$, the matching pair (*i*, *j*) is not a blocking pair, regardless of the values of the other remaining variables.

If $x_{ij}=0$, when b=1, i.e., ∑j′<jixij′=0, The logistics provider *i* has no matching worse than client *j*, and the matching pair (*i*, *j*) must not be a blocking pair. When b=0, i.e., ∑j′<jixij′=1. The logistics provider *i* matches at least one client that is worse than *j*, then,

When 0≤c, i.e., $\underset{i^{\prime }{>}_{j}i}{\sum }x_{i^{\prime }j}W_{i^{\prime }}+W_{i}\geq W_{j}{}^{'}$, if client *j* and logistics provider *i* form a match, the total load of the matched logistics provider exceeds the weight of the total goods of client *j*. Therefore, client *j* will not reach a new match with logistics provider *i*. The matching pair (*i*, *j*) is not a blocking pair.

When −1<c<0, i.e., $0<\underset{i^{\prime }{>}_{j}i}{\sum }x_{i^{\prime }j}W_{i^{\prime }}+W_{i}<W_{j}{}^{'}$, it means that client *j* is willing to form a new match with logistics provider *i*, and the matching pair (*i*, *j*) is a blocking pair.

In summary, the matching pair (*i*, *j*) is a blocking pair if and only if flag<0. Therefore, constraint (38) can ensure that no blocking pairs exist. Based on the preference matrices ***A*** and ***B*** of the provider and client, the logistics−client many-to-one stable matching model is constructed as follows:


$$\text{maximum}\;z_{1}=\overset{m}{\underset{i=1}{\sum }}\overset{n}{\underset{j=1}{\sum }}x_{ij}a_{ij}$$ (39)

$$\text{maximum}\;z_{2}=\overset{m}{\underset{i=1}{\sum }}\overset{n}{\underset{j=1}{\sum }}x_{ij}b_{ji}$$ (40)

$$\text{minimum}\;z_{3}=\overset{m}{\underset{i=1}{\sum }}\overset{n}{\underset{j=1}{\sum }}x_{ij}\left|a_{ij}-b_{ji}\right|$$ (41)

$$\text{s}\text{.t}\text{.}\;\overset{n}{\underset{j=1}{\sum }}x_{ij}\leq 1,\;\forall i$$ (42)

$$\overset{m}{\underset{i=1}{\sum }}x_{ij}W_{i}\leq W_{j}{}^{'},\;\forall j$$ (43)

$$x_{ij}\left[\left(\frac{b_{j}{}^{'}+b_{i}}{2}-c_{i}\right)d_{j3}W_{i}-c_{i}\left(d_{ij2}+d_{ji4}\right)\right]\geq \text{0, }\forall i,j$$ (44)

$$x_{ij}\frac{\left(d_{ij2}+d_{ji4}\right)}{d_{i1}+d_{ij2}+d_{j3}+d_{ji4}}\leq \gamma \text{, }\forall i,j$$ (45)

$$x_{ij}\left(b_{j}{}^{'}-b_{i}\right)\geq 0,\forall i,j$$ (46)

 xij+1−∑j′<jixij′+∑i′>jixi′jWi′+Wi−Wj'Wj'≥0, ∀i,j (47)

$$x_{ij}\in \left\{0,1\right\},\;\forall i,j$$ (48)

The first objective function (39) maximizes the overall preference of logistics providers with respect to clients.

The second objective function (40) maximizes the overall preference of clients with respect to logistics providers.

The third objective function (41) minimizes the overall preference deviation to achieve overall fairness.

Constraints (42) show that one logistics provider can only be matched to one client.

Constraints (43) express that the total load of logistics vehicles matched to a client cannot exceed the total weight of the client’s goods.

Constraints (44) indicate that the earnings of logistics providers should be greater than zero.

Constraints (45) show that the empty-loaded rate of any logistics provider is not more than *γ*.

Constraints (46) show that the cost savings of clients should be greater than zero.

Constraints (47) represent a stable matching to avoid blocking pairs.

Constraints (48) show that the value of the decision variable is 0 or 1.

Similar to the objective function in the logistics−client one-to-one stable matching problem in Section 4.3, the three objective functions are also converted to a single objective function.


$$\text{maximum}\;\overset{m}{\underset{i=1}{\sum }}\overset{n}{\underset{j=1}{\sum }}x_{ij}a_{ij}b_{ji}$$ (49)

## 5 Experiment study

This section analyzes two examples of the logistics−client stable matching problems in one-to-one and many-to-one situations, both with the 500 ×  200 (logistics−client) data scale. The hardware environment is a mainframe with “Windows 10, 16 G memory, 2.7 GHz processor”. The data were randomly generated.

### 5.1 Logistics−client one-to-one stable matching case

MATLAB was used to randomly generate truncated normal distribution data of the related variables. The mean, standard deviation, and value ranges of the variables are shown in Table 3.

**Table 3:** Setting of indices and parameters of one-to-one stable matching.

Indices	Value range	(mean and standard deviation)
*m*	500	/
n	200	/
Parameters		
$d_{i1}$	5−700	(350, 50)
$d_{ij2}$	5−30	(20, 5)
$d_{j3}$	5−700	(350, 50)
$d_{ij4}$	5−30	(20, 5)
$W_{i}$	5−80	(50, 5)
$V_{i}$	3−90	(70, 10)
$W_{j}^{'}$	5−80	(40, 5)
$V_{j}^{'}$	3−90	(60, 10)
$v_{i}$	40−100	(60, 20)
$c_{i}$	1−6	(3, 0.8)
$s_{i}$	0−5	(3, 1)
$b_{i}$	3−8	(5, 0.8)
$b_{j}^{'}$	5−10	(7, 0.8)

**Fig 2:**
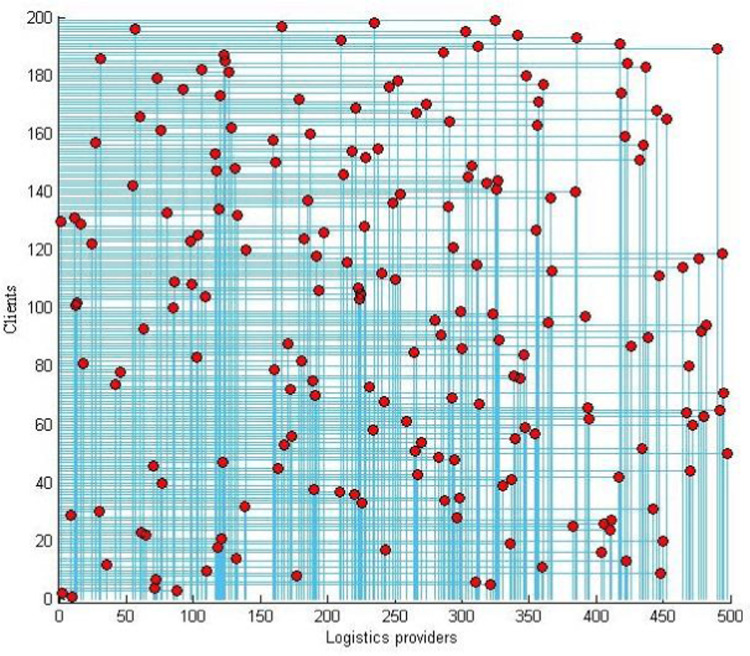
One-to-one matching result chart of 500 ×  200 scale.

The case has 500 ×  200 =  100,000 variables and 700,700 constraints. By using the Cplex linear solver, the program outputs the global optimal objective value as 59.2856, with a running time of 632.709 s. The optimal solution has 198 stable matching pairs of logistics providers and clients, as shown in Fig 2.

**Fig 3:**
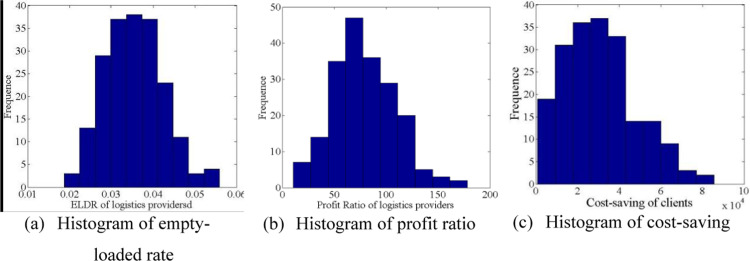
Histogram of serval index.

The red points indicate the points where the logistics providers of the corresponding columns match with the clients, while the blue lines indicate the exact values of the corresponding points on the two axes. The histograms of the three indices are shown in Fig 3 to illustrate the effectiveness of the logistics−client one-to-one stable matching model.

The empty-loaded rate histogram of the matched logistics providers is shown in Fig 3 (a), where most empty-loaded rates fall between 0.035 and 0.0375, and all empty-loaded rate values are within 6%. The histogram of the profit ratio of the matched logistics providers shows that most values fall between 60 and 80. Fig 3(c) shows that most cost savings of clients are within [2, 4].

**Fig 4:**
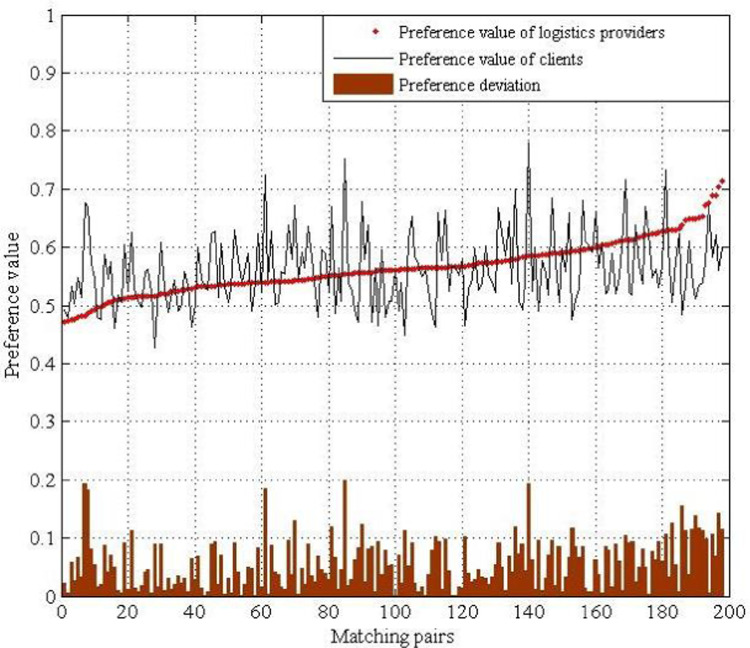
Statistical chart of preference deviation of the logistics providers and clients.

Next, we analyzed the preference deviation between the matched logistics−client pairs, as shown in Fig 4. The preference values of the logistics providers are sorted in ascending order, and the preference values of matched clients are represented by a black line. The bar shows that the preference deviations of all matched pairs are within 0.2.

### 5.2 Logistics−client many-to-one stable matching case

This section presents a case study on the logistics−client many-to-one stable matching problem with a scale of 500 ×  200. First, MATLAB was used to randomly generate truncated normal distribution data of the variables, as shown in Table 4.

**Table 4:** Setting of indices and parameters of many-to-one stable matching.

Indices	Value range	(Mean and standard deviation)
*M*	500	/
*N*	200	/
Parameters		
$d_{i1}$	5−700	(350, 100)
$d_{ij2}$	5−30	(20, 5)
$d_{j3}$	5−700	(350, 100)
$d_{ij4}$	5−30	(20, 5)
$W_{i}$	5−80	(40, 20)
$W_{j}^{'}$	500−1500	(1000, 200)
$v_{i}$	40−100	(60, 20)
$c_{i}$	1−8	(3, 0.8)
$s_{i}$	0−5	(3, 1)
$b_{i}$	3−10	(5, 0.8)
$b_{j}^{'}$	3−10	(7, 0.8)

**Fig 5:**
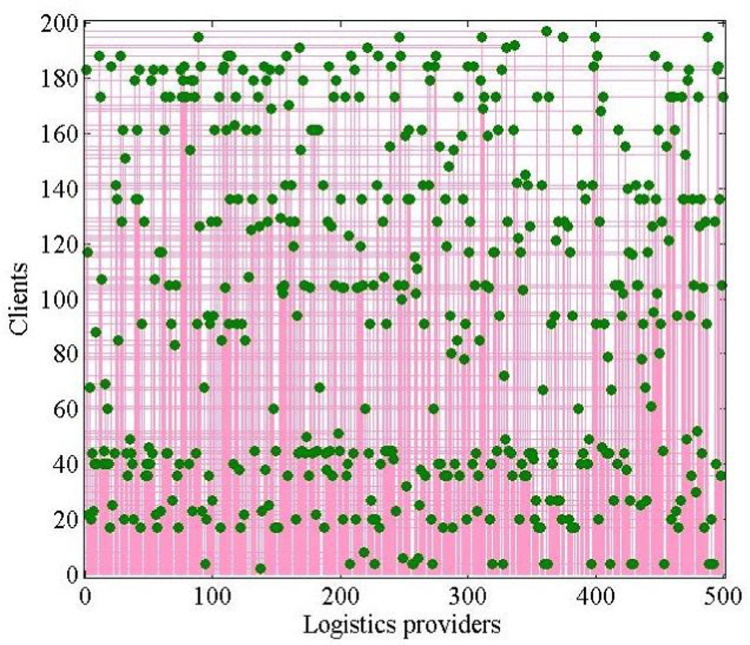
Many-to-one matching result chart of 500 ×  200 scale.

Using the same linear solver of Cplex as in Section 5.1, the 500 ×  200 scale many-to-one stable matching problem was tested. The problem has 100,000 variables and 500,700 constraints. The program outputs the global optimal objective value as 137.3157, with 447.6602 s running time. The optimal logistics−client many-to-one stable matching solution is shown in Fig 5.

**Fig 6:**
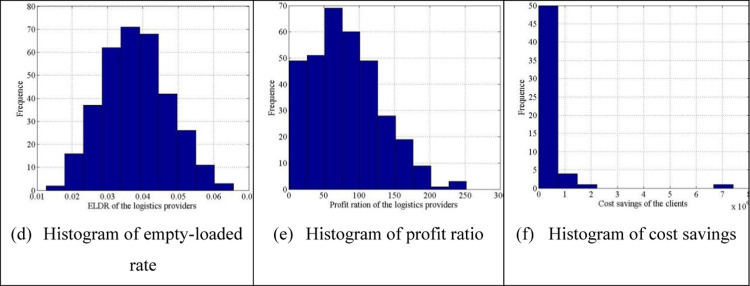
Histogram of serval index.

In Fig 5, there are 338 matching pairs, where green points indicate that the points where the logistics providers of the corresponding rows match with the clients, while the pink lines indicate the exact values of the corresponding points on the two axes. In many-to-one stable matching, one logistics provider matches with only one client, but one client can match with multiple logistics providers to obtain sufficient delivery services for the bulk goods. The histograms of the three indices of the optimal solution are shown in Fig 6.

According to the segmentation interval of the above histograms, most empty-loaded rates fall between 0.03 and 0.04, and all empty-loaded rate values are within 7%. The histogram of the profit ratio of logistics providers shows that most values fall between 50 and 75. Fig 6(f) shows that most cost savings of clients are within [0, $1\times 10^{6}$]. In many-to-one stable matching, the cost-saving histogram is more uneven when compared to that of one-to-one stable matching.

**Fig 7:**
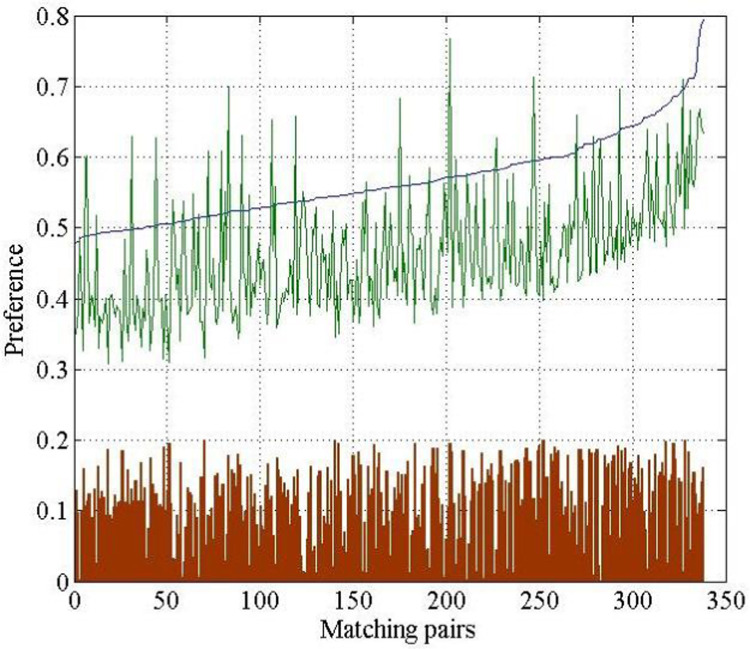
Statistical chart of preference deviation of the logistics provider and clients.

Next, we analyze the preference deviation between the logistics−client matching pairs, as shown in Fig 7. The preference values of the logistics providers are sorted in ascending order, preference values of matched clients are drawn in a line, and the bar in Fig 7 illustrates that the deviations of all matching pairs are within 0.2.

## 6 Case study

To further verify the effectiveness of the proposed models, this section presents a real case study based on the open-source real data on “CSIT-China Road Freight Electronic Trading Platform (https://www.zczy56.com/common/sourceOfGoods)” (October 5, 2019). CSIT is a logistics trading sharing platform that provides goods source information and vehicle source information registered on the platform.

### 6.1 Data description and processing

The original delivery routes of the logistics providers and demand delivery routes of the client are obtained by crawling the open-source data on the CSIT platform. The data that have the same departure and destination sites and incomplete information were removed. There are 112 remaining logistics delivery routes and 147 remaining demand delivery routes. The data styles of logistics providers and clients in the main cities of China are shown in Tables 5 and 6 as an example. The specific calculation process is as follows: 1) locate the longitude and latitude coordinates of local names on the Baidu map, and 2) by accessing the Baidu Map API, the vehicle driving distance between two coordinates with high speed and priority is obtained.

**Table 5:** Original transportation route data of logistics provider.

Number	Type of vehicle	Load (ton)	Departure site	Destination site	Distance (km)	Type of goods	Weight (ton)	Start time	End time	Cost (Yuan/ton/km)	Charge (Yuan/ton/km)	Average Assessment score [1–5]
1	Flatbed truck	70	Anyang City, Henan Province	Jining City, Shandong Province	386.32	Electronic yarn	70	2019/6/13 17:25	2019/6/13 18:25	9.30	9.849,54	4.55
2	Flatbed truck	70	Anyang City, Henan Province	Zibo City, Shandong Province	449.14	Electronic yarn	70	2019/6/13 17:07	2019/6/13 18:07	9.30	9.785,229	3.90
3	Flatbed truck	70	Zibo City, Shandong Province	Anyang City, Henan Province	452.06	Bobbin	70	2019/6/13 17:22	2019/6/13 18:22	9.30	10.190,48	4.6
4	High hurdle truck	70	Chongqing City	Xianyang City, Hubei Province	832.19	Home appliances	70	2019/6/13 23:00	2019/6/13 23:59	9.30	10.098,96	4.64
…	…	…	…	…	…	…	…	…	…	…	…	
112	Flatbed truck	40	Changzhi city, Shanxi province	Yanan City, Shanxi Province	413.44	Rebar	30	2019/6/13 13:33	2019/6/14 12:59	10.20	10.370,43	3.81

**Table 6:** Transport demand data of client.

Number	Departure site	Destination site	distance (km)	Type of goods	Weight (ton)	Start time	End time	Bid (Yuan/ton/km)
1	Huaian City, Jiangsu Province	Xinyu City, Jiangxi Province	854.97	Chemical industry	20	2019/3/22 16:29	2019/3/23 16:26	8.28
2	Nanjing City, Jiangsu Province	Taizhou City, Jiangsu Province	186.84	Rebar	42.5	2019/6/30 13:32	2019/6/30 17:45	10.75
3	Ningbo City, Zhejiang Province	Suzhou City, Jiangsu Province	179.09	Bearing	1.4	0	0	6.53
4	Ningbo City, Zhejiang Province	Qinzhou City, Guangxi Autonomous Region	2,021.08	Injection molding machine	28	2019/8/25 22:46	2019/8/26 12:46	8.55
	…	…	…	…	…	…	…	…
147	Luoyang City, Henan Province	Yuncheng City, Shanxi Province	142.65	Steel Pipe	13.50	2019/11/10 13:20	2019/11/10 17:00	7.42

The two ends of the line represent the departure and destination sites of the logistics providers and clients. There are more distribution orders in eastern China than in western China. This is because the eastern region is more developed in terms of the economy and transportation than that of the western region. According to the data obtained from the official website of the CSIT platform, most transported goods are mineral products. The place with the most orders for transportation is Chenzhou City in Hunan Province, which is referred to as the “hometown of non-ferrous metals”. The second place with the most orders for transportation is Tangshan City in Hebei Province, which has rich mineral resources.

**Fig 8:**
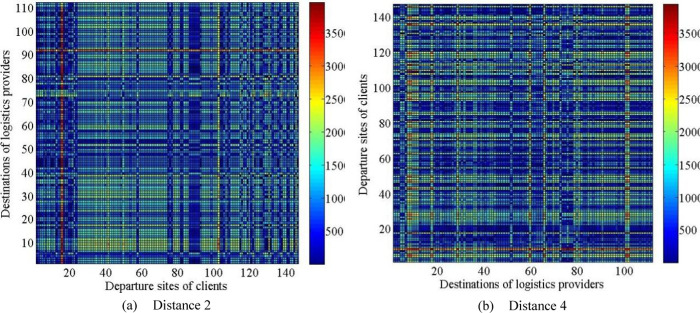
Histogram of serval index.

Based on the above data, the distance 2 between the destinations of logistics providers and departure sites of clients and the distance 4 between the destinations of clients and the original departure sites of logistics providers are calculated, as shown in Fig 8.

Then, the preference matrix *A* of logistics providers with respect to clients and the preference matrix *B* of clients with respect to logistics providers can be calculated according to the above data. It is not displayed here because each matrix has a large variable scale of 500×200=100000.

### 6.2 Result of one-to-one matching

Based on the preference matrices *A* and *B*, we optimized the logistics−client one-to-one stable matching using the CPLEX tool. The optimal objective value is 10.5914, with the corresponding optimal matching results for the main cities in China.

**Fig 9:**
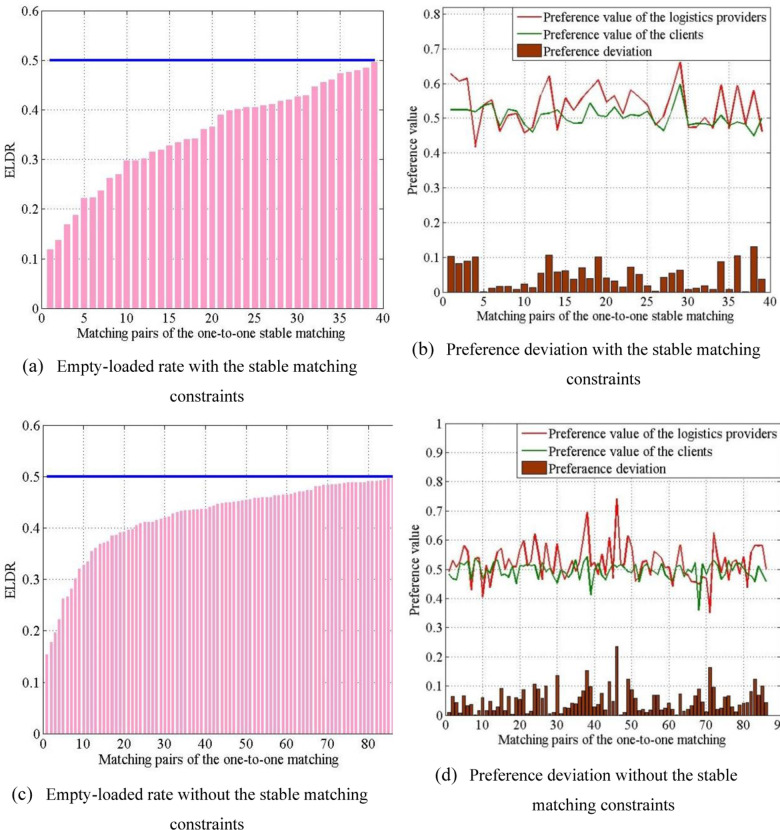
Matching result analysis of the one-to-one matching.

Next, we analyzed the empty-loaded rate and preference deviation of matching pairs in the one-to-one matching, as shown in Fig 9. Two situations with and without stable matching constraints were compared with each other.

Fig 9(a) shows that the empty-loaded rate of all matching pairs is lower than 0.5. This illustrates that the model is effective in reducing the empty-loaded rate when compared to the conventional logistics pattern, which has an empty-loaded rate of 0.5. Fig 9(b) shows the logistics−client matching preference values and the preference deviations between them. All matched preference deviations were within 0.15.

The result without the stable matching constraints is shown in Fig 9(c) and (d). The empty-loaded rate and preference deviation without stable matching constraints were higher than those with stable matching constraints. In other words, the average matched empty-loaded rates with and without the stable matching constraints were 0.3535 and 0.4244, respectively, and the average matched preference deviations with and without the stable matching constraints were 0.0461 and 0.0509, respectively.

The comparison indicates that in the empty-loaded rate and preference deviation, the logistics−client one-to-one matching model with the stable matching constraints performs better than the other one.

### 6.3 Result of many-to-one matching

In this section, we study a real case of the logistics−client many-to-one stable matching problem in which clients have bulk goods. The data source was mined from the bulk quantities of goods on the CSIT platform, where 28 groups of bulk goods data (including route 3) were selected, as shown in Table 7. The original delivery data (route 1) of the logistics vehicle are the same as those described in Section 6.1. Routes 2 and 4 were calculated according to the corresponding coordinates.

**Table 7:** Delivery demand data of client’s bulk goods.

Number	Departure site	Destination site	Distance (km)	goods	Weight (ton)	Bid (yuan/ton/km)
1	Linfen City, Shanxi Province	Jincheng City, Shanxi Province	178.37	Water quenching	520	12.90
2	Yulin City, Shanxi Province	Xingtai City, Hebei Province	716.13	Surface coal	1,000	18.10
3	Tianjin City	Wulanchabu City, Inner Mongolia Autonomous Region	548.9	Oman ore	500	18.10
4	Xinzhou City, Shanxi Province	Jincheng City, Shanxi Province	462.62	Iron ore fines	998.76	18.10
5	Yanan City, Shanxi Province	Xuchang City, Henan Province	677.66	coal	5,000	17.89
…	…	…	…	…	…	…
28	Lianyungang City, Jiangsu Province	Jincheng City, Shanxi Province	767.38	Iron ore	98,888	13.48

We used the linear optimizer embedded in the Cplex tool to optimize the logistic−client many-to-one stable matching problem with the linear planning model constructed in Section 4.4. The optimal objective value is 23.2017.

**Fig 10:**
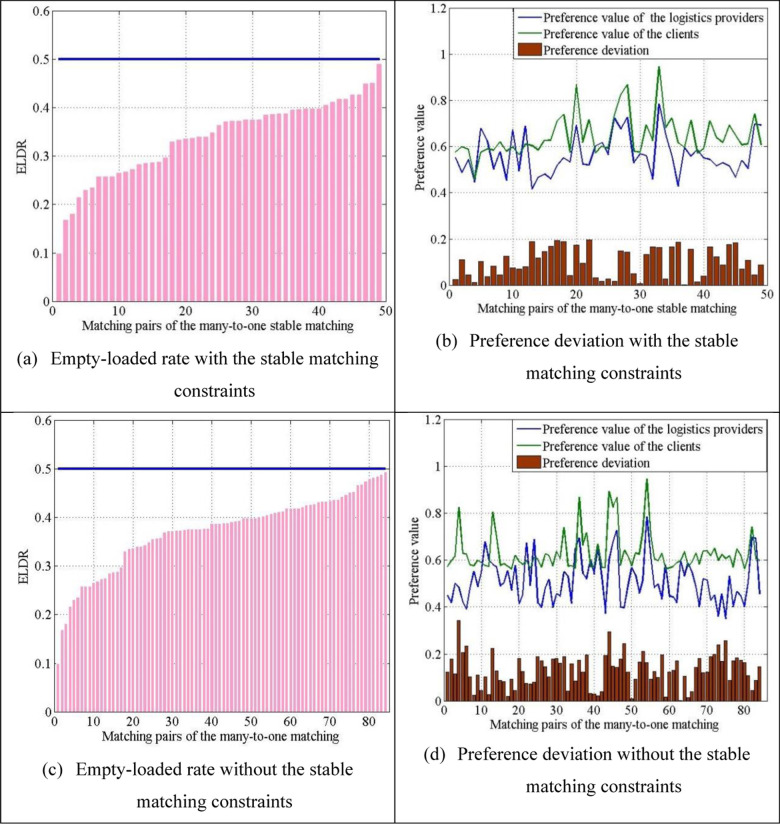
Matching result analysis of the many-to-one matching.

Then, as presented in the previous section, we compare the empty-loaded rate and preference deviation of matching pairs under two situations: with and without the many-to-one stable matching constraints. The comparison results are presented in Fig 10.

Fig 10(a) and (c) are the empty-loaded rates with and without stable matching constraints. The empty-loaded rate of the two cases was within 0.5, as empty−loaded rate<0.5 is a basic constraint of the planning model. However, the average matched empty-loaded rate without the stable matching constraints was 0.3729, which is larger than the average matched empty-loaded rate with the stable matching constraints of 0.3391.

Fig 10(b) and (d) show the preference values between logistics providers and clients and the preference deviations between them under the aforementioned two cases. The average matched preference deviations with and without the stable matching constraints were 0.09892 and 0.1297, respectively.

The comparison indicates that in the empty-loaded rate and preference deviation, the logistics−client many-to-one matching model with the stable matching constraints performs better than that without the stable matching constraints.

## 7 Discussion and conclusion

This study encompassed separate experiments and case studies. The experimental study involved the random generation of data for 500 logistics providers and 200 customers. In the one-to-one scenario, we established 198 matched pairs, while in the many-to-one scenario, 338 matched pairs between 56 customers and 338 logistics providers were formed, affirming the viability of the matching model. The experimental study demonstrates the applicability of the proposed one-to-one and many-to-one models to a large-scale problem involving 100,000 variables. We contend that larger-scale problems can be addressed with improved hardware. In the case study, utilizing real-world data, we observed that in the one-to-one matching, the majority of empty running rates fell within the range of [0.46, 0.5]. Conversely, in the many-to-one matching scenario, the majority of empty running rates fell within the range of [0.339, 0.419], indicating the potential for logistics providers serving multiple customers to reduce empty running rates. Notably, in both scenarios, matched logistics providers experienced increased revenue, while customers realized cost reductions.

In conclusion, this study addresses the prevalent issue of high empty-loaded rates in conventional transportation patterns. Real-world delivery demands often fall into two categories: small quantities of goods and bulk quantities of goods. To mitigate empty loads during return trips, this study develops stable one-to-one and many-to-one matching models between logistics providers and clients, employing linear planning to support efficient and global optimization. Specifically, for logistics-client many-to-one stable matching, we introduce innovative constraints to address the variability in the number of logistics providers required by clients, which depends on the total capacity of the matched logistics providers.

A primary contribution of this study lies in the formulation of a linear one-to-one and many-to-one stable matching model for logistics-client relationships, including the establishment of novel linear many-to-one stable matching constraints. However, the real case study also indicates that stringent stable constraints reduce the number of matching pairs. Future research may explore relaxing these stable constraints to enhance the matching ratio.

While this article proposes matching models considering individual fairness, overall fairness, and stability, additional research avenues are identified. Specifically, future investigations could explore stable matching problems under incomplete preference lists and non-strict ordering, relax stability constraints to enhance match success rates and reduce empty running rates, and conduct sensitivity analyses for individual fairness threshold parameters. These potential research directions hold promise for advancing the applicability and effectiveness of matching models within the logistics domain, offering opportunities for further improvements in addressing transportation efficiency and fairness considerations.
